# Phyllodes tumors of the breast: Real world data from a multi-institution cohort

**DOI:** 10.1016/j.breast.2025.104491

**Published:** 2025-05-06

**Authors:** Carl Sars, Jan Frisell, Paul W. Dickman, Felix Haglund de Flon, Fredrik Karlsson, Helena Sackey, Ebba K. Lindqvist

**Affiliations:** a Department of Molecular Medicine and Surgery, Karolinska Institutet, Stockholm, Sweden; b Division of Cancer, Department of Breast, Endocrine Tumors and Sarcoma, Karolinska University Hospital, Stockholm, Sweden; c Department of Medical Epidemiology and Biostatistics, Karolinska Institutet, Stockholm, Sweden; d Department of Oncology-Pathology, Karolinska Institutet, Stockholm, Sweden; e Department of Clinical Science and Education, Stockholm South General Hospital, Karolinska Institutet, Stockholm, Sweden; f Department of Surgery, Stockholm South General Hospital, Stockholm, Sweden

## Abstract

**Introduction:**

Phyllodes tumors (PT) are rare breast lesions arising from fibroepithelial stroma and may be hard to clinically distinguish from fibroadenomas. They are defined as benign, borderline or malignant. The purpose of this study was to describe diagnostic workup and surgical management, and to investigate incidence of local recurrence (LR) and overall survival (OS) in relation to tumor subtype, size, age, surgical margins, surgical method, and year of diagnosis.

**Methods:**

Retrospective cohort study of all patients surgically treated for a PT in Stockholm, Sweden from 1999 to 2018. Descriptive analyses were performed, and regression models were used to analyze associations between selected covariates and LR and OS.

**Results:**

Among 191 patients, 132 were treated for a benign PT, 40 for a borderline PT and 19 for a malignant PT. Preoperatively, results from diagnostic workup were often ambiguous, and only 45.6 % of cases had a preoperative diagnosis of PT. Initial surgery was breast-conserving in 93.2 % of patients. Recurrences occurred in 10.5 % of the total cohort. 5-year and 10-year OS was 96.1 % and 93.5 %, respectively, for the entire cohort. In a multivariable analysis, neither covariate was associated with risk of LR. Distant recurrences were only detected among patients with malignant PT.

**Conclusions:**

In the workup of PT, common diagnostic methods such as FNAC, CNB, and mammography may be unreliable, and clinical suspicion plays a critical role in guiding pre-operative decision-making. We found no association between surgical margins and rate of LR or OS. We found no evidence of metastatic potential in benign or borderline PT.

## Highlights

• Breast tumor diagnostic methods like FNAC, CNB and mammography may be unreliable in ruling out phyllodes tumors.
• Clinical suspicion of a phyllodes tumor plays a critical role in guiding pre-operative decision-making.
• No apparent association between surgical margins and rate of local recurrence or overall survival in phyllodes tumors.
• This study found no evidence of metastatic potential in benign or borderline phyllodes tumors.

## 1 Introduction

Phyllodes tumors (PT) are rare fibroepithelial tumors arising in the breast and are classified as benign, borderline, or malignant [1,2]. Although benign PT do not exhibit the ability to metastasize or become malignant, all types of PT may recur locally [3,4]. PT may be hard to distinguish from other mesenchymal tumors, including fibroadenomas, and consensus on the diagnosis, management and follow-up is lacking [[5], [6], [7], [8]]. Moreover, the long-standing recommendation that a wide excision is necessary to avoid local recurrence (LR) in PT has recently been challenged, especially for benign PT where it is no longer recommended [[9], [10], [11], [12]]. The National Comprehensive Cancer Network guideline recommendation of a routine clinical follow-up for three years for all individuals with PT appears to be without strong evidence [12].

Recent efforts to map the current clinical landscape have shown considerable variation in the clinical management of PT [[13], [14], [15]]. Thus, currently clinicians lack robust evidence on reliable pre-operative diagnostic measures, appropriate surgical method or margin and long-term real-world data on clinically significant outcomes such as local recurrence rates and mortality.

In lieu of prospective, randomized data, the clinical management of rare conditions must be based on high-quality, observational, real-world data. Previous publications are in general single-center studies, and most reports include only patients who were registered in specific databases which might introduce selection bias [16,17]. Sweden has a single-payer healthcare system, producing few barriers to healthcare delivery. We herein present long-term data from a multi-institution regional cohort in a low-threshold, publicly funded, decentralized healthcare system that offers universal healthcare for all its residents. We sought to provide new aspects on the clinical management and long-term outcomes of these tumors with minimal risk of selection bias or loss to follow-up.

The purpose of this study was to describe diagnostic workup and surgical management of PT, and to investigate overall survival (OS) and rate of local recurrence (LR) in relation to tumor subtypes, size, surgical margins, surgical method, age, and year of diagnosis.

## 2 Methods

### 2.1 Design

This was a retrospective cohort study of all patients who underwent surgery with curative intent at any hospital in the Stockholm region, Sweden, for a primary PT during the study period January 1, 1999 to December 31, 2018, with follow-up for all outcomes until June 30th, 2021. Potentially eligible patients were identified by searching the regional, Karolinska-based electronic medical database for histopathological codes, Systematized Nomenclature of Medicine (SNOMED), which includes all cases of cancer in the Stockholm region that are diagnosed by a histopathologist. The search for PT of the breast was performed by combining codes for topography (C50 breast and C49 connective tissue, subcutaneous tissue and other soft tissue) and morphology (phyllodes: 90201/b; 90203, angiosarcoma 91203, atypical liposarcoma 88501/b, carcinosarcoma 89803, fibromyxosarcoma 88113, fibrosarcoma 88103, hemangiosarcoma 91203, Kaposi sarcoma 91403, clear cell sarcoma 90443, chondrosarcoma 92203, leiomyosarcoma 88903, lymphangiosarcoma 91703, myosarcoma 88953, myxosarcoma 88403, pleomorphic cell sarcoma 88023, sarcoma NOS 88003, small cell sarcoma 88033 and synovial sarcoma 90433). This broad search was made to ensure we did not miss any misclassified cases, as malignant PT may be hard to distinguish from other sarcoma. Each case was cross-checked for a match between SNOMED code and diagnosis on final histopathology report by a histopathologist and electronic medical records (EMR) were then reviewed to confirm the diagnosis and treatment of PT, other tumor types were discarded. A pre-operative biopsy was not required, as final diagnosis was based on the surgical specimen. Three exclusion criteria were applied to increase the internal validity of the study: those individuals who during the study period had surgery for a recurrent tumor with the primary resection being carried out before the study period; those whose tumors were primarily located on the thorax and not in the breast; and those who were operated without curative intent. Thus, the final study cohort consisted of patients with a primary PT of the breast who had surgery with curative intent during the study period.

The study was approved by the Ethical Review Board of Stockholm, 2020/03335 and 2021/02924. The manuscript was prepared according to the STROBE (Strengthening the reporting of observational studies in epidemiology) Statement guidelines (https://www.strobe-statement.org).

### 2.2 Data collection

All study variables, that is exposure, outcomes, and covariates, were collected and reviewed from EMR as described below. Patient characteristics and medical management data were collected and included age, sex, smoking status, previous medical history including breast tumors, previous irradiation, comorbidities, ASA class, clinical workup, date of diagnosis of PT, date and method of treatment, follow-up protocol, and details on the primary tumor such as localization, histological subtypes, size, and surgical margins from histopathological reports. Information on patient ethnicity was not available for the cohort. Clinical outcomes, including incidence and location of recurrence, and OS were ascertained through manual review of EMR. Outcome for OS was available for all patients due to automatic linking of EMR to the national population registry. Outcome for LR and distant recurrence (DR) was administratively censored at last contact for those who migrated from the Stockholm region.

### 2.3 Exposures

The study exposure in the analysis of long-term outcomes was the different PT subtypes, defined as benign, borderline or malignant. This approach was similar to previous studies examining local recurrence rates and overall survival [9,18].

### 2.4 Outcomes

Main study outcome was a descriptive analysis of diagnostic workups and surgical treatment methods. Secondary outcomes were incidence of local recurrence and overall survival, analyzed for all tumor types and as well as separately analyzed for each subtype, and in association to select covariates (see below).

### 2.5 Covariates

Five potential confounders were considered when analyzing LR rate (with categorization in brackets); age (continuous variable), tumor size (continuous variable), year of diagnosis (categorical variable in 5-year intervals), final resection margin status (negative, R0 or positive, R1), and surgical method (breast conserving or mastectomy).

Resection margin status was defined as negative (tumor-free [R0]) or positive (microscopic or macroscopic tumor involvement [R1 or R2]) as exact resection margin was not available for all cases and because there is no other established definition of clear margins in these tumors [[13], [14], [15]]. During data collection, the surgical intent of the primary resection was often not apparent from the EMR notes, unless a mastectomy was performed. This is likely due to the inherent difficulties of establishing a pre-operative diagnosis in PT. Therefore, it was not possible to consistently separate lumpectomy with curative intent from excisional biopsy with diagnostic intent. Lumpectomy and excisional biopsy were thus grouped together as breast conserving surgery (BCS), which was compared against mastectomy as a binary variable. We then focused on whether or not clinicians were satisfied with the margins of excision or opted for a second resection.

Additionally, a sixth confounder, comorbidity (categorized score 0, I or ≥ II according to the most well-validated version of the Charlson comorbidity index, not counting the current breast tumor) [19] was considered when analyzing OS.

### 2.6 Statistical analysis

Patient demographics, clinical workup, treatment, and tumor characteristics were summarized and described using numbers (n) and frequencies (%). For continuous variables, the median and interquartile range were calculated and presented alongside the range.

Using a Cox proportional hazards model, univariable and multivariable models were fitted to calculate hazard ratios (HR) to estimate the effect of selected covariates (surgical margins, tumor size, age, surgical method, and year of diagnosis as a categorical variable) on the relative hazard of LR and additionally co-morbidity on the relative hazard of death (OS). Patients with missing information on any covariates in the models were excluded (n = 24) leaving 167 patients (87.4 %) eligible for complete case analysis in the final model for the secondary outcomes. No imputation was done for missing values. Time since date of first surgery was the underlying time scale. Tests of the proportional-hazards assumption based on scaled Schoenfeld residuals showed no evidence against the proportional hazards assumption. Unadjusted survival proportions were estimated using the Kaplan-Meier (KM) method and compared using the log-rank test. Patients were followed until time of event, end of study (administrative censoring), or until loss to follow-up. Median follow-up time was calculated using reverse KM estimate.

Results are reported using 95 % confidence intervals (CI) and p-values, the significance level was 0.05. Statistical analyses were performed using Stata/BE 17 (StataCorp LP, Texas, USA).

## 3 Results

### 3.1 Patient characteristics

We identified 191 women with a PT treated at seven institutions within the Stockholm region during the study period. Median tumor size was 35 mm (interquartile range [IQR] 24–55 mm) with a range of 1–320 mm. On final histopathological diagnosis, tumors were classified as benign in 69.1 % (n = 132), borderline in 20.9 % (n = 40) and malignant in 10.0 % (n = 19). Median age at diagnosis was 46 years (IQR 38–58 years). The majority were never smokers (56.3 %, n = 107) and had no history of benign breast tumors (e.g., fibroadenoma) (63.9 %, n = 122) and had no history of breast cancer (96.3 %, n = 184) (Table 1).

**Table 1:** Patient demographics, by phyllodes tumor subtype, 1999–2018.

	Overall n = 191	Benign PT n = 132	Borderline PT n = 40	Malignant PT n = 19
Age at diagnosis, median (IQR)	46 (38–58)	44 (36–57)	50 (43–61)	50 (42–62)
Year of diagnosis, n (%)				
1999–2003	25 (13.1)	18 (13.6)	3 (7.5)	4 (21.1)
2004–2008	41 (21.5)	36 (27.3)	4 (10.0)	1 (5.3)
2009–2013	62 (32.5)	42 (31.8)	12 (30.0)	8 (42.1)
2014–2018	63 (33.0)	36 (27.3)	21 (52.5)	6 (31.6)
Tumor size in mm, median (IQR)	35 (24–55)	30 (1–120)	45 (7–320)	47 (21–180)
Laterality, n (%)				
Left	97 (50.8)	64 (48.5)	21 (52.5)	12 (63.2)
Right	94 (49.2)	68 (51.5)	19 (47.5)	7 (36.8)
Bilateral	0 (0.0)	0 (0.0)	0 (0.0)	0 (0.0)
Smoking status, n (%)				
Current	23 (12.0)	17 (12.9)	4 (10.0)	2 (10.5)
Previous	14 (7.3)	7 (5.3)	5 (12.5)	2 (10.5)
Never	107 (56.0)	74 (56.1)	23 (57.5)	10 (52.6)
Unknown	47 (24.6)	34 (25.8)	8 (20.0)	5 (26.3)
ASA class, n (%)				
1	133 (69.6)	96 (72.7)	26 (65.0)	11 (57.9)
2	48 (25.1)	31 (23.5)	12 (30.0)	5 (26.3)
3	9 (4.7)	5 (3.8)	1 (2.5)	3 (15.8)
4	1 (0.5)	0 (0.0)	1 (2.5)	0 (0.0)
History of previous benign breast tumor				
None	122 (63.9)	79 (59.8)	29 (72.5)	14 (73.7)
Ipsilateral	49 (25.7)	36 (27.3)	9 (22.5)	4 (21.1)
Contralateral	13 (6.8)	12 (9.0)	1 (2.5)	0 (0.0)
Bilateral	7 (3.7)	5 (3.8)	1 (2.5)	1 (5.3)
History of previous breast cancer				
None	184 (96.3)	127 (96.2)	38 (95.0)	19 (100.0)
Ipsilateral	3 (1.6)	2 (1.5)	1 (2.5)	0 (0.0)
Contralateral	4 (2.1)	3 (2.3)	1 (2.5)	0 (0.0)
Bilateral	0 (0.0)	0 (0.0)	0 (0.0)	0 (0.0)
Previous radiation therapy to the thoracic wall				
Yes	3 (1.6)	1 (0.8)	0 (0.0)	2 (10.5)
No	188 (98.4)	131 (99.2)	40 (100.0)	17 (89.5)

### 3.2 Preoperative evaluation and diagnosis

For the majority of patients, initial presentation of the PT was a palpable breast lesion (74.9 %, n = 143). Most patients (84.3 %, n = 161) underwent a pre-operative fine-needle aspiration cytology (FNAC). Results from the FNAC report were diverse (Supplementary Table 1). Only 61 patients (31.9 %) underwent a pre-operative CNB. Diagnostic workup included mammography in 72.3 % (n = 138) of cases whereof 22.0 % (n = 42) were BI-RADS code 1 or 2. A multi-disciplinary team conference (MDT), took place in 89.5 % of cases, either pre- and/or postoperatively. In less than half of cases (45.6 %, n = 187) there was a pre-surgical clinical suspicion of PT or sarcoma according to notes in the medical record (Table 2).

**Table 2:** Diagnostic workup by Phyllodes Tumor Subtype, 1999–2018.

	Overall n = 217	Benign PT n = 132 (60.1 %)	Borderline PT n = 40 (18.4 %)	Malignant PT n = 19 (8.8 %)
Mammography code (BI-RADS), n (%)				
1	2 (1.0)	1 (0.8)	1 (2.5)	0 (0.0)
2	40 (20.9)	29 (22.0)	9 (22.5)	2 (10.5)
3	61 (31.9)	42 (31.8)	11 (27.5)	8 (42.1)
4	28 (14.7)	19 (14.4)	6 (15.0)	3 (15.8)
5	7 (3.7)	3 (2.3)	4 (10.0)	0 (0.0)
Missing	53 (27.7)	38 (28.8)	9 (22.5)	6 (31.6)
Initial presentation				
Screening	43 (22.5)	33 (25.0)	8 (20.0)	2 (10.5)
Palpable lump	143 (74.9)	95 (72.0)	31 (77.5)	17 (89.5)
Unknown	5 (2.6)	4 (3.0)	1 (2.5)	0 (0.0)
FNAC				
No	30 (15.7)	13 (9.8)	11 (27.5)	6 (31.6)
Yes	161 (84.3)	119 (90.2)	29 (72.5)	13 (68.4)
CNB				
No	130 (68.1)	100 (75.8)	20 (50.0)	10 (52.6)
Yes	61 (31.9)	32 (24.2)	20 (50.0)	9 (47.4)
MDT				
None	16 (8.4)	16 (12.1)	0 (0.0)	0 (0.0)
Pre-operative	28 (14.7)	21 (15.9)	6 (15.0)	1 (5.3)
Post-operative	43 (22.5)	29 (22.0)	10 (25.0)	4 (21.1)
Both	100 (52.4)	63 (47.7)	23 (57.5)	14 (73.7)
Unknown/missing	4 (2.1)	3 (2.3)	1 (2.5)	0 (0.0)
Preliminary pre-operative diagnosis^a^				
Benign/Fibroadenoma	90 (47.1)	70 (53.0)	16 (40.0)	4 (21.1)
Suspected phyllodes/sarcoma	87 (45.6)	53 (40.2)	22 (55.0)	12 (63.2)
Atypia/malignancy NOS (diagnostic biopsy)	7 (3.7)	4 (3.0)	1 (2.5)	2 (10.5)
Breast cancer	7 (3.7)	5 (3.8)	1 (2.5)	1 (5.3)

Abbreviations: NOS, Not otherwise specified. MDT, Multidisciplinary Team meeting/Tumor board. FNAC, Fine-needle aspiration cytology. CNB, Core-needle biopsy.

a According to medical records from pre-operative assessment and/or MDT.

### 3.3 Operative management and adjuvant treatment

Initial operative management was BCS in 93.2 % (n = 178) and mastectomy in 6.8 % (n = 13) of patients. Few patients (5.8 %, n = 11) underwent axillary surgery, whereof n = 8 underwent a sentinel lymph node biopsy (SLNB) and n = 3 underwent axillary lymph node dissection (Table 3).

**Table 3:** Overview of surgical and adjuvant treatment by Phyllodes Tumor Subtype, 1999–2018.

	Overall n = 191	Benign PT n = 132 (69.1 %)	Borderline PT n = 40 (20.9 %)	Malignant PT n = 19 (9.5 %)
Primary surgical treatment				
Breast Conserving Surgery	178 (93.2)	129 (97.7)	37 (92.5)	12 (63.2)
Mastectomy	13 (6.8)	3 (2.3)	3 (7.5)	7 (36.8)
Surgical margins after first operation				
Negative	123 (64.4)	88 (66.7)	25 (62.5)	10 (52.6)
Positive	58 (30.4)	36 (27.3)	14 (35.0)	8 (42.1)
Unknown	10 (5.2)	8 (6.1)	1 (2.5)	1 (5.3)
Surgical margins after second operation				
Negative	154 (80.6)	101 (76.5)	36 (90.0)	17 (89.5)
Positive	27 (14.1)	23 (17.4)	3 (7.5)	1 (5.3)
Unknown	10 (5.2)	8 (6.1)	1 (2.5)	1 (5.3)
Sentinel lymph node biopsy (SLNB)				
Yes	8 (4.2)	2 (1.5)	2 (5.0)	4 (21.1)
No	183 (94.5)	130 (98.5)	38 (95.0)	15 (78.9)
Axillary lymph node dissection				
Yes	3 (1.6)	2 (1.5)	0 (0.0)	1 (5.3)
No	188 (98.4)	130 (98.5)	40 (100.0)	18 (94.7)
Second operation performed				
Breast Conserving Surgery	31 (16.2)	13 (9.8)	15 (37.5)	3 (15.8)
Mastectomy	9 (4.7)	1 (0.8)	2 (5.0)	6 (31.6)
Additional wide excision on thoracic wall (post-mastectomy)	2 (1.0)	0 (0.0)	0 (0.0)	2 (10.5)
None	149 (78.0)	118 (89.4)	23 (57.5)	8 (42.1)
Residual tumor burden∗				
Yes	7 (16.7)	1 (7.1)	4 (23.5)	2 (18.2)
No	35 (83.3)	13 (92.9)	13 (76.5)	9 (81.8)
Adjuvant treatment	4 (2.1)	1 (0.8)	1 (2.5)	2 (10.5)
Radiation therapy (RT)	3 (1.6)	1 (0.8)	1 (2.5)	1 (5.3)
Chemotherapy (CT)	0 (0.0)	0 (0.0)	0 (0.0)	0 (0.0)
RT + CT	1 (0.5)	0 (0.0)	0 (0.0)	1 (5.3)
Endocrine therapy	1 (0.5)	0 (0.0)	1 (2.5)	0 (0.0)
None	188 (98.4)	131 (99.2)	39 (97.5)	17 (89.5)

Abbreviations: RT, radiation therapy. CT, chemotherapy. ∗ Percentage of those who had second surgery.

After the initial operation, surgical margin status was negative (complete surgical resection with tumor-free margins [R0]) in 64.4 % (n = 123), positive (intralesional [R1]) in 30.4 % (n = 58) and unknown (due to missing data) in 5.2 % (n = 10, whereof n = 8 benign, n = 1 borderline and n = 1 malignant). Out of the 58 cases with an initial positive margin, a second operation was performed in 14 out of 36 benign, 11 out of 14 borderline, and 7 out of 8 malignant PT. In total, a second surgical resection was performed in 22.0 % (n = 42) of the entire cohort including 10 patients with initial negative margins. This second operation included an additional wide excision of the thoracic wall in n = 2, repeated BCS in n = 31, and mastectomy in n = 9. Residual disease was identified in seven patients (16.7 %) who underwent a second operation (Table 3). In total, the final surgical margins among those who underwent a second operation, were negative in all but one case, which was a benign PT. Surgical margins remained positive without a second operation in 23 benign, 3 borderline and 1 malignant PT.

The vast majority of patients (97.4 %) did not receive any adjuvant treatment. Three women (1.6 %) received adjuvant radiation therapy, one (0.5 %) both radiation therapy and chemotherapy, and one (0.5 %) endocrine therapy.

### 3.4 Local and distant recurrence

Median follow-up time for recurrences were 10.1 years (95 % CI 8.64 to 11.14). Recurrences occurred in 10.5 % of the total cohort (n = 18 LR, n = 2 DR) during the study period. LR occurred in 7.6 % (n = 10) of benign PT, 17.5 % (n = 7) of borderline PT and 5.6 % (n = 1) of malignant PT. The two DR occurred in 10.5 % (n = 2) of malignant PT (Table 4).

**Table 4:** LR incidence by Final Margin Status and Phyllodes Tumor Subtype, 1999–2018.

Negative margins				
	Overall n = 154	Benign n = 101	Borderline n = 36	Malignant n = 17
LR cases	15	9	5	1
LR incidence rate	9.7 %	8.9 %	13.9 %	5.9 %

**Positive margins**				
	**Overall n = 27**	**Benign n = 23**	**Borderline n = 3**	**Malignant n = 1**

LR cases	2	1	1	0
LR incidence rate	7.4 %	4.3 %	33.3 %	0.0 %

Abbreviations: LR, local recurrence. PT, phyllodes tumor.

LR occurred in 7.4 % (n = 2) of cases with positive margins and 9.7 % (n = 15) of cases with a negative margin and 10 % (n = 1) patient with unknown margin status which was not a statistically significant difference in incidence (p = 0.70). LR occurred at a median of 26.6 months (95 % CI, 19.1 to 35.91) while DR occurred after 23.2 and 49.5 months, respectively.

### 3.5 Survival

**Fig. 1:**
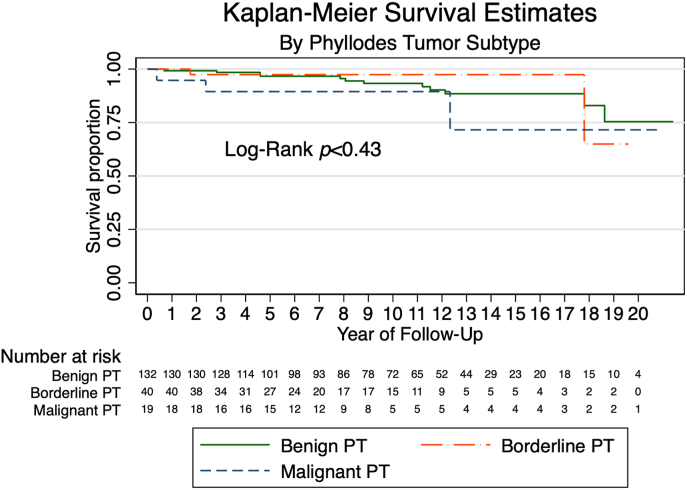
Survival Proportions for Overall Survival by Phyllodes tumor subtype. Abbreviations: PT, phyllodes tumor.

Median follow-up time for overall survival was 10.1 years (95 % CI 9.11 to 11.37). Seventeen patients (8.9 %) died during follow-up, of which 7 died within 5 years of diagnosis. Patients with malignant PT had the highest frequency of death (n = 3/19, 15.8 %). 5-year overall survival in the cohort was 96.1 % (95 % CI 91.9 to 98.1). OS subdivided by tumor type is summarized in Table 5. Estimates of OS for each tumor type are shown in Fig. 1.

**Table 5:** Long-term overall survival by phyllodes tumor subtype, 1999–2018.

	Overall n = 191	Benign n = 132	Borderline n = 40	Malignant n = 19
**5-year (95 % CI)**	96.1 % (91.9–98.1)	96.6 % (91.3–98.7)	97.1 % (80.9–99.6)	88.9 % (62.4–97.1)
**10-year (95 % CI)**	93.5 % (88.1–96.5)	93.3 (86.4–96.8)	97.1 % (80.9–99.6)	88.9 % (62.4–97.1)

Estimated crude and adjusted HRs from Cox proportional hazards models for selected covariates are shown in Supplementary Tables 2 and 3 We used uni- (crude) and multivariable (adjusted) Cox regression to estimate HRs for LR and OS with selected covariates. Year 2009–2013 of diagnosis, as compared to index years 1999–2003 (adjusted HR 14.23, 95 % CI 1.13 to 177.84, p = 0.04) and mastectomy, as compared to BCS, (adjusted HR 6.20, 95 % CI 1.48 to 26.02, p = 0.01) were associated with higher risk of mortality. No association was found between covariates and risk of LR. We found no statistically significant association between final margin status and LR or death.

## 4 Discussion

In this multi-institutional regional cohort study, including 191 women with all subtypes of 10.13039/100023865PT of the breast, we describe real-world management and long-term clinical outcomes within a low-threshold, publicly funded decentralized healthcare system. We found that in the workup of PT, common diagnostic methods such as FNAC, CNB, and mammography may be unreliable, and were not consistently used. Thus, clinical suspicion plays a critical role in guiding pre-operative decision-making. Positive surgical margins were not found to be associated with risk of LR. In benign and borderline PT, we found a high OS and no evidence of distant recurrence over a long follow-up time.

The definitive diagnosis of PT occurs after surgical excision and is often unexpected due to the diagnostic uncertainty of mammography, FNAC and CNB. In our cohort, the rate of pre-operative FNAC was generally high (84.3 %) but had limited clinical value as made evident by the broad range of cytological findings (Supplementary Table 1). Conversely, the rate of pre-operative CNB was lower (31.9 %) but provided less diverse pathology reports (not presented) which may have a higher clinical value. This is similar to previous reports on CNB in diagnosing PT, which suggest higher positive predictive value in CNB than in FNAC [5,20,21].

Our results indicate that both FNAC and CNB have not always been used regularly and may provide false negative results, which implies that a clinical suspicion of PT is of high importance pre-operatively, and that inconclusive or benign results on pre-operative biopsies cannot be relied upon to rule out PT when clinically suspected. FNAC was found to have limited diagnostic utility for PTs, possibly due to its inability to assess tissue architecture critical for distinguishing these lesions from fibroadenomas. This limitation may have resulted in diagnostic inaccuracies and suboptimal initial management [22]. We therefore recommend against the use of FNAC as a standalone diagnostic tool in suspected cases of PT. Since only a smaller fraction underwent a CNB, we did not dive deeper into the diagnostic accuracy of those biopsies because of the risk of selection bias. We cannot speculate on why some patients underwent a FNAC and other a CNB as this data is not available. The National Comprehensive Cancer Network (NCCN) guidelines state that in certain instances, distinguishing between a fibroadenoma and a PT may prove challenging with FNAC or CNB(12). While CNB exhibits higher sensitivity in diagnosing PT compared to FNAC, neither method consistently discerns PT from fibroadenomas [12](12). Newer diagnostic approaches for cytology shows promising results to distinguish cancer cells from normal ones and correlates well to Nottingham score in breast cancer cells [23] and may be useful in diagnosing fibroepithelial lesions in the future.

Over the course of the study period, diagnostic practices evolved, with CNB increasingly replacing FNAC as the preferred method for evaluating fibroepithelial lesions. This shift likely improved diagnostic precision by allowing better assessment of stromal architecture, critical for distinguishing PTs from fibroadenomas. Thus, the 10.13039/100013700NCCN recommends that when clinical suspicion arises regarding a 10.13039/100023865PT, excision of the lesion may be necessary for an accurate pathological classification, and our results support this recommendation. A topic for future research could be to explore the possible impact of a known diagnosis pre-operatively compared to a post-operative diagnosis on the rate of re-excision and long-term outcomes.

Notably, similar rates of BI-RADS score 4 were detected among benign (14.4 %), borderline (15.0 %) and malignant (15.8 %) PT. Furthermore, a few benign PT were graded as BI-RADS score 5 (highly suspicious for malignancy) and most noteworthy, 10.5 % of malignant PT were graded as benign (BI-RADS score 2). These findings indicate that mammography alone is not a reliable method for identifying these tumors or their malignancy potential. Our results suggests that when a clinical suspicion of PT arises, low emphasis should be placed on pre-operative biopsies and mammography. We did not collect data on indication for surgery in cases with a low BI-RADS or negative biopsy, although we speculate that in some cases, it might have been a presumed fibroadenoma (with symptoms of pain) or a clinical suspicion of PT e.g. fast growth.

We found an unexpectedly high rate (4.2 %) of SLNB in our cohort (highest, 21.1 %, among patients with malignant PT), and notably two patients with benign PT underwent axillary lymph node dissection. Although we do not have information on the indications for axillary surgery among the patients in our cohort, we speculate that axillary surgery may have been performed because of indeterminate pre-operative workup, where for example concurrent adenocarcinoma could have been suspected. Adjuvant radiation therapy use was scarce, which is to be expected for low grade tumors. The use of adjuvant radiation therapy in our cohort was lower in malignant PT compared to its use in malignant PT in a report from the Surveillance, Epidemiology, and End Results Program (SEER) database [16]. We speculate that the potential benefit of radiation therapy in malignant PT has gained interest only in the recent years.

BCS was performed for most benign and borderline PT, with a higher rate of mastectomy among malignant PT. Bearing in mind their excellent long-term outcomes, our results show that both benign and borderline PT can be safely managed with BCS. Treatment approaches for PTs have also evolved internationally over the past two decades. There has been a trend toward standardized surgical management, altough margin widths is still a topic of debate. While mastectomy was previously more commonly performed for larger tumors, current practice increasingly supports breast-conserving surgery when adequate margins can be achieve. Considering the difficulty of distinguishing benign, borderline and malignant PT in the pre-operative workup, we suggest that BCS should always be the first choice when PT is suspected, if it is deemed possible to achieve safe margins considering the size of the lesion in relation to the size of the breast.

In 30.4 % of cases surgical margins were positive after primary surgery, with the highest frequency among benign PT. The residual tumor burden in re-excision specimens was found to be relatively small (16.7 %). We cannot in our data differentiate between wide or narrow margins, due to unreported exact margins.

Rates of recurrence (10.5 %) and of LR (7.6 %) were low and similar regardless of final surgical margin status. The two DR occurred within five years of surgery. Although our findings must be interpreted with caution due to the small sample size, our results do not support the value of clear margins when it comes to avoiding 10.13039/501100009319LR in 10.13039/100023865PT. These findings are in concordance with several previous studies that have investigated the relationship between margin status and recurrence in various subtypes of PT [9,11,24]. Current guidelines from the NCCN recommends excisional biopsy in benign PT, and at least 10 mm margin in borderline and malignant PT (12). In recent years a number of investigators have sought to answer the same research question; what surgical margin is sufficient in different subtypes of PT? Several observational studies conclude that narrow or positive surgical margin may increase the risk of LR, favoring the notion to always seek wide excisional margins [[25], [26], [27], [28], [29], [30], [31]]. On the contrary, many reports indicate that the rate of LR is not influenced by the degree of surgical margins [[9], [10], [11],^24^,[32], [33], [34], [35], [36], [37], [38], [39]]. Two meta-analyses on the topic have conflicting conclusions on the impact of margins on the LR rate [40,41]. The LR rate in the present study was lower (8.9 %) for malignant PT as compared to contemporary large cohorts, reported at 11.7–12.5 % [9,18]. This could be due to smaller sample size, differences in patient presentation or surgical strategy. Newer evidence from a large Dutch national cohort suggests low LR rates but found no associations with negative margins and LR for borderline and malignant PT (18). In contrast, the same group found a positive surgical margin to be associated with recurrence in benign PT [42]. We found no association between final margin status and increased risk of LR or increased risk of mortality when adjusting for potential confounders in our cohort.

These results would warrant an updated meta-analysis in light of newer published reports, or international efforts for a randomized clinical trial.

Importantly, no instance of DR occurred in benign or borderline PT. This finding has implications on protocols for clinical and radiological follow-up, and may suggest that intermittent radiological screening, for example with chest computed tomography, and initial radiological staging, is of limited value in benign and borderline PT.

We report long-term OS similarly favorable for benign and borderline PT, yet as expected, worse for malignant PT. Previous studies have reported similar survival rates for borderline and malignant PT [28], 43, 44).

Our study exhibits several strengths, as it encompasses a large cohort of PT spanning over two decades, with little loss to follow-up. 10.13039/100014337Furthermore, the data is from a multi-institution cohort in a publicly funded healthcare setting with few barriers to care. Our database contains all histopathological samples from our region, ensuring that we could capture all cases, even those benign or borderline that are not routinely reported to national cancer registries. Rigorous investigation of patients’ EMR provided granular data and automatic linkage to national registries of migration and/or death. These factors contributed to ensure minimal missing data on outcomes, compared to studies in other settings where loss to follow-up is more frequent and patients with recurrences may not be identified. Limitations of our study include the retrospective observational setting where some data, such as exact surgical margins and indications for axillary surgery, were not available. It cannot be excluded that one or two patients may have undergone surgical treatment influenced by an incorrect pre-operative diagnosis of breast cancer, which might have resulted in narrower initial surgical margins (Supplementary Table 1). Data on use of pre-operative ultrasound or pre-operative MRI was unfortunately not collected for this study. Furthermore, due to ambiguity in the EMR notes we were unable to separate excisional biopsy with diagnostic intent from lumpectomy with curative intent. Since these two procedures might have differing margin intents, enucleation vs. attempt at a negative margin, grouping them together as BCS may have masked true effects. The timespan of the cohort ensures a long follow-up for many of the included patients but may not reflect contemporary management for the patients included in the early years of the cohort. Finally, low event rates and a limited sample size made estimations from our multivariable analysis uncertain.

To conclude, our results based on real-world data from this cohort of individuals with a PT of the breast demonstrate diagnostic challenges and increases the emphasis on clinical reasoning in cases of suspected PT. Based on our findings, we recommend against using FNAC in initial diagnostics. The role of adequate surgical margins to avoid LR in PT remains unproven. Long-term outcomes for benign and borderline PT are favorable, which has implications on patient-directed information as well as on follow-up protocols. In the future, clinician-scientists should focus on collaborative efforts to investigate the optimal surgical margins for different subtypes of PT, and to determine the most effective modality and frequency of follow-up after treatment.

## CRediT authorship contribution statement

**Carl Sars:** Writing – review & editing, Writing – original draft, Methodology, Investigation, Formal analysis, Data curation, Conceptualization. **Jan Frisell:** Writing – review & editing, Validation, Supervision, Methodology, Investigation, Formal analysis, Conceptualization. **Paul W. Dickman:** Writing – review & editing, Supervision, Software, Resources, Methodology, Investigation. **Felix Haglund de Flon:** Writing – review & editing, Methodology, Investigation, Formal analysis, Conceptualization. **Fredrik Karlsson:** Writing – review & editing, Methodology, Investigation, Formal analysis. **Helena Sackey:** Writing – review & editing, Supervision, Methodology, Investigation, Formal analysis, Conceptualization. **Ebba K. Lindqvist:** Writing – review & editing, Supervision, Methodology, Investigation, Funding acquisition, Formal analysis, Data curation, Conceptualization.

## Declaration of competing interest

The authors declare the following financial interests/personal relationships which may be considered as potential competing interests:

Carl Sars reports financial support was provided by Magnus Bergvall Foundation. If there are other authors, they declare that they have no known competing financial interests or personal relationships that could have appeared to influence the work reported in this paper.
