# Supplementary material for: Phyllodes tumors of the breast: Real world data from a multi-institution cohort
Source: Breast. 2025 May 6;82:104491. doi: 10.1016/j.breast.2025.104491 (PMC12282485; doi:10.1016/j.breast.2025.104491)
Supplement: Multimedia component 1 [file mmc1.docx]

**Supplementary Table 1**

|  | **Overall  n=191** | **Benign  n=132** | **Borderline  n=40** | **Malignant  n=19** |
| --- | --- | --- | --- | --- |
| Adenocarcinoma | 1 |  | 1 |  |
| Atypia NOS | 3 | 3 |  |  |
| Atypical epithelial cells | 4 | 2 | 1 | 1 |
| Benign ductal epithelia | 1 | 1 |  |  |
| Benign fatty tissue | 1 | 1 |  |  |
| Benign hyperplastic mammary epithelia | 5 | 2 | 3 |  |
| Benign mammary epithelial cells | 12 | 10 | 1 | 1 |
| Benign NOS | 5 | 4 |  | 1 |
| Breast cancer | 1 | 1 |  |  |
| Epithelial proliferation | 1 | 1 |  |  |
| Fatty tissue/fatty necrosis | 1 | 1 |  |  |
| Fibroadenoma | 68 | 54 | 13 | 1 |
| Fibroadenoma or phyllodes | 14 | 14 |  |  |
| Fibroepithelial tumor with atypia | 1 |  | 1 |  |
| Giant cell fibroma | 1 | 1 |  |  |
| Giant fibroadenoma or phyllodes | 2 | 1 |  | 1 |
| Insufficient material | 2 |  | 1 | 1 |
| Macrophages, no malignancy | 1 |  | 1 |  |
| Malignant epithelial cells | 2 |  | 1 | 1 |
| Mesenchymal tumor/sarcoma | 2 |  |  | 2 |
| Mucinous breast cancer | 1 | 1 |  |  |
| Papillary tumor | 3 | 3 |  |  |
| Papilloma with atypia | 1 | 1 |  |  |
| Phyllodes NOS | 21 | 16 | 4 | 1 |
| Phyllodes, benign | 2 |  | 2 |  |
| Phyllodes, malignant | 2 |  |  | 2 |
| Pleomorphic adenoma | 1 | 1 |  |  |
| Sarcoma NOS | 1 |  |  | 1 |
| Tumor NOS | 1 | 1 |  |  |
|  |  |  |  |  |
| Total | 161 | 119 | 29 | 13 |

**FNAC report on cytologic findings by final post-operative histopathology diagnosis.** Abbreviations: FNAC, Fine-needle aspiration cytology. NOS, Not otherwise specified

**Supplementary Table 2**

| **Assessed variable** | **Crude** |  |  |  | **Adjusted** |  |  |
| --- | --- | --- | --- | --- | --- | --- | --- |
|  | **HR** | **p-value** | **95% CI** |  | **HR** | **p-value** | **95% CI** |
| **Age (continuous)** | 1.09 | <0.001 | 1.05-1.13 |  | 1.06 | 0.08 | 0.99-1.15 |
| **Charlson comorbidity score** |  |  |  |  |  |  |  |
| 0 | 1.00 (ref) | — | — |  | 1.00 (ref) | — | — |
| I | 1.42 | 0.68 | 0.28-7.24 |  | 1.12 | 0.93 | 0.11-11.65 |
| ≥II | 9.81 | <0.001 | 2.73-35.19 |  | 2.29 | 0.51 | 0.53-27.08 |
| **Year of diagnosis** |  |  |  |  |  |  |  |
| 1999-2003 | 1.00 (ref) | — | — |  | 1.00 (ref) | — | — |
| 2004-2008 | 3.33 | 0.27 | 0.39-28.51 |  | 8.04 | 0.10 | 0.69-93.81 |
| 2009-2013 | 5.02 | 0.15 | 0.57-44.54 |  | 14.23 | 0.04 | 1.13-177.84 |
| 2014-2018 | 3.60 | 0.33 | 0.28-47.14 |  | 5.92 | 0.28 | 0.24-146.80 |
| **Surgical treatment** |  |  |  |  |  |  |  |
| BCS | 1.00 (ref) | — | — |  | 1.00 (ref) | — | — |
| Mastectomy | 3.94 | 0.02 | 1.25-12.46 |  | 6.20 | 0.01 | 1.48-26.02 |
| **Surgical margins** |  |  |  |  |  |  |  |
| Negative | 1.00 (ref) | — | — |  | 1.00 (ref) | — | — |
| Positive | 0.94 | 0.93 | 0.21-4.20 |  | 3.03 | 0.21 | 0.53-17.28 |
| **Tumor size (continuous)** | 1.01 | 0.32 | 0.99-1.02 |  | 0.99 | 0.51 | 0.98-1.01 |

**Estimated crude and adjusted HRs from Cox proportional hazards models for selected covariates on the risk of death.** Abbreviations: HR, hazard ratio. BCS, breast conserving surgery

**Supplementary Table 3**

| **Assessed variable** | **Crude** |  |  |  | **Adjusted** |  |  |
| --- | --- | --- | --- | --- | --- | --- | --- |
|  | **HR** | **p-value** | **95% CI** |  | **HR** | **p-value** | **95% CI** |
| **Age (continuous)** | 0.97 | 0.08 | 0.94-1.00 |  | 0.98 | 0.19 | 0.94-1.01 |
| **Year of diagnosis** |  |  |  |  |  |  |  |
| 1999-2003 | 1.00 (ref) | — | — |  | 1.00 (ref) | — | — |
| 2004-2008 | 0.41 | 0.33 | 0.07-2.47 |  | 0.41 | 0.38 | 0.57-3.00 |
| 2009-2013 | 0.97 | 0.97 | 0.21-4.45 |  | 0.83 | 0.84 | 0.13-5.14 |
| 2014-2018 | 1.85 | 0.41 | 0.43-7.93 |  | 1.58 | 0.60 | 0.28-8.85 |
| **Surgical treatment** |  |  |  |  |  |  |  |
| BCS | 1.00 (ref) | — | — |  | 1.00 (ref) | — | — |
| Mastectomy | 0.00 | 1.00 | 0 |  | 0.00 | 1.00 | 0 |
| **Surgical margins** | 0.78 | 0.74 | 0.18-3.42 |  | 0.70 | 0.64 | 0.16-3.11 |
| Negative | 1.00 (ref) | — | — |  | 1.00 (ref) | — | — |
| Positive |  |  |  |  |  |  |  |
| **Tumor size (continuous)** | 1.00 | 0.13 | 1.00-1.02 |  | 1.02 | 0.08 | 1.00-1.04 |

**Estimated crude and adjusted HRs from Cox proportional hazards models for selected covariates on the risk of local recurrence.** Abbreviations: HR, hazard ratio. BCS, breast conserving surgery
